# Some Diseases of the Male Genital System

**Published:** 1908-03-28

**Authors:** 


### POINTS IN SURGERY.

### SOME DISEASES OF THE MALE GENITAL SYSTEM.

### XII. MALIGNANT DISEASE OF THE TESTIS.

MALIGNANT disease attacking the testis is one of the most virulent forms of new growth. This is contrary to expectation, for one might suppose that in an organ which is isolated, as the testicle is, the surgeon would have a good chance of eradicating the disease by a thorough removal of the organ and all its annexes in the early stages, better, for instance, than he would have in dealing with carcinoma in an organ like the breast, which has a complicated system of lymphatic vessels draining into adjacent glands. Unhappily this is not the case; whether this be due to the fact that malignant disease of the testis is not readily recognised in its earlier stages, or because the lymphatic glands which are enlarged are situated in an inconspicuous position it is not possible to say.

We have been, and still are, accustomed to recognise two main varieties of malignant disease attacking the testis—carcinoma and sarcoma; the former being said to occur in middle age and the latter in young adult life. An attempt has recently been made to prove that many cases of so-called sarcoma of the testis are in reality carcinomatous. The argument rests on the assertion that, if successive sections of a sarcomatous testis are cut, it is often found that in some part of the specimen the cells approach the epithelial type, and, more than this, they present an alveolar arrangement. This is an interesting pathological speculation, but it does not yet rest on a sufficiently solid basis to be accepted as an established fact, and, in any case, it is not of much clinical importance, since, even if it is true, it does not modify the line of treatment which should be adopted in any given case.

Carcinoma starts, as far as we know, in the epithelium lining the seminal tubules. It forms a rapidly-growing swelling in the centre of the testis, and causes primarily a uniform ovoid enlargement of the organ. It is only later when the tunica albuginea is invaded and gives way that its surface becomes irregular. If left to pursue its course unchecked it often undergoes a mucoid degeneration, and the neighbouring parts, epididymis and tunica vaginalis, are eventually involved. After an interval, which is usually not a long one, the lumbar glands become enlarged, and from these deposits may extend into almost any abdominal viscus.

We know nothing, of course, of the causation of carcinoma of the testis, and all the time-honoured theories have been put forward in turn to account for it. But one cannot pass the subject by without referring to Cohnheim's ingenious idea that the growths originate in fœtal relics which exist in the testis and lie latent for years. He suggests that the cells in these relics suddenly start to grow at a later period. But why should they do this? What is the stimulus which excites them into activity? That is the question which must be solved before we can make any headway in our knowledge of malignant disease in general. For in all malignant disease we are dealing with cells of the body which have started to multiply, without regard to the needs of the body, in response to some unknown stimulus.

Even if we do not accept the hypothesis mentioned above that many of the so-called sarcomata of the testis are in reality carcinomatous there seems little doubt that sarcoma is less common than carcinoma. When it occurs it starts in the connective tissue elements of the organ. Its clinical course resembles that of carcinoma very closely. It causes at first a uniform enlargement of the organ, which progresses rapidly, and after a time its surface becomes irregular. As in carcinoma the lumbar lymphatic glands are enlarged. One commonly reads that sarcoma is spread by blood-vessels and carcinoma by the lymphatic system. It is not possible to make any such dogmatic statement; one not infrequently finds sarcoma involving the lymphatic glands, apart from the text-book examples of sarcoma of the tonsil, testis and thyroid; and conversely how can one explain the presence of nodules of carcinoma in the liver secondary to disease of the rectum except on the ground that metastasis takes place by means of the portal vein? All that one can say is that malignant disease spreads by the nearest and most convenient channel, and in the case of a sarcoma, which often possesses blood-sinuses lined only by a single layer of endothelium, this channel is undoubtedly the blood-stream.

Since the only chance of saving the life of a patient afflicted with malignant disease of the testis is to remove the organ early (and at the best that chance is not a very good one) it is of the utmost importance to be able to diagnose the condition in its early stages. Tuberculous disease may generally be recognised by the fact that the epididymis is affected before the body of the testis and by the thickening of the cord. More difficulty may be experienced with syphilitic orchitis. In both the disease comes on in adult life, and in both it starts typically as a uniform enlargement of the body of the testis, and further in both a history of past syphilis may be obtainable. This last may be more misleading, since it is a natural assumption to suppose that in a patient who gives a definite history of syphilis an enlargement of the testicle is due to that disease. Lastly, malignant disease of the testis is sometimes associated with free fluid in the tunica vaginalis and may be mistaken for a hæmatocele. Aspiration of the tunica will solve the difficulty. In sarcoma the fluid withdrawn is bright red and may have the consistency of pure blood, whereas in hæmatocele it is generally altered so as to be chocolate brown in colour.

The safest rule is to suspect all rapidly growing tumours of the testis, and, if the diagnosis cannot be made certain by clinical methods, to remove a piece for microscopical examination. Should the disease prove to be malignant the testis should be removed widely and at once; but it is useless to do this when once the lumbar glands are enlarged so as to be palpable through the abdominal wall.

